# Effects of different speed-accuracy instructions on perception in psychology experiments: evidence from event-related potential and oscillation

**DOI:** 10.3389/fnins.2024.1354051

**Published:** 2024-05-31

**Authors:** Haijian Li, Xiaoshuang Wang, Timo Hamalainen, Zhaoli Meng

**Affiliations:** ^1^School of Sport and Health Sciences, Dalian University of Technology, Dalian, China; ^2^Faculty of Information Technology, University of Jyväskylä, Jyväskylä, Finland; ^3^School of Biomedical Engineering, Faculty of Medicine, Dalian University of Technology, Dalian, China

**Keywords:** perceptual process, speed-accuracy trade-offs, event-related potentials, event-related oscillation, principal component analysis

## Abstract

**Introduction:**

In cognitive behavioral experiments, we often asked participants to make judgments within a deadline. However, the most common instruction of “do the task quickly and accurately” does not highlight the importance of the balance between being fast and accurate.

**Methods:**

Our research aimed to explore how instructions about speed or accuracy affect perceptual process, focus on event-related potentials (ERPs) and event-related oscillations (EROs) of two brain responses for visual stimuli, known as P1 and N1. Additionally, we compared the conventional analysis approach with principal component analysis (PCA) based methods to analyze P1 and N1 ERP amplitude and ERO power.

**Results:**

The results showed that individuals instructed to respond quickly had lower P1 amplitude and alpha ERO than those who prioritized accuracy, using the PCA-based approach. However, these two groups had no differences between groups in the N1 theta band using both methods. The traditional time-frequency analysis method could not detect any ERP or ERO distinctions between groups due to limitations in detecting specific components in time or frequency domains. That means PCA is effective in separating these components.

**Discussion:**

Our findings indicate that the instructions given regarding speed and accuracy impact perceptual process of subjects during cognitive behavioral experiments. We suggest that future researchers should choose their instructions carefully, considering the purpose of study.

## 1 Introduction

When making decisions, people often face time constraints that require them to determine when to stop thinking and start choosing. Sometimes, individuals feel the need to evaluate a situation carefully. For instance, when taking an exam, sticking to the deadline for submitting your paper is essential. Similarly, when you see a traffic light turning red, it is crucial to hit the brakes. These examples show that decision-making involves finding a balance between being prompt in responding and being accurate in selecting. This balance is commonly referred to as the speed-accuracy trade-off (SAT), where making decisions faster usually means sacrificing some level of accuracy and vice versa (Duckworth et al., 2017; Ranger et al., 2021).

There has been a growing interest within the neuroscience community regarding the SAT for years. Neurological investigations of the SAT have helped us better understand executive function, decision-making processes, and behavioral regulation in humans (Heitz, 2014). Individuals aware of the scoring system’s response time component may try to optimize their SAT. Moreover, psychological decision-making experiments involve ambiguous instructions, requiring subjects to respond quickly and accurately (Yu et al., 2020). However, it has been observed that subjects may interpret instructions differently, resulting in varied choices on the SAT (Liesefeld and Janczyk, 2019). This variability in SAT choices among subjects within a group may have influenced the results. Therefore, experimenters need to be fully aware of each subject’s choices on the SAT (Bogacz et al., 2010). However, most current methods rely on the subjectivity of the participants. Researchers are now increasingly taking notice of this problem. Furthermore, thanks to advancements in brain imaging tools over the years, there is potential to explore the neural mechanisms underlying different choices made on the SAT.

Many psychological studies have attempted to use brain imaging tools to investigate the impact of trade-offs between speed and accuracy on brain signals. Some studies that used fMRI have discovered that subjects had increased blood oxygen level-dependent (BOLD) imaging when asked to respond quickly (Forstmann et al., 2008; Ivanoff et al., 2008; Bogacz et al., 2010). Specifically, Ho et al. (2012) used fMRI to explore whether reduced performance under speed stress indicates suboptimal information processing in areas like the visual cortex (V1). In their experiment, participants performed a task involving judgment of orientation while focusing either on response speed or accuracy. Their study revealed that the rate at which perceptual evidence accumulates is selectively influenced when individuals prioritize precision but not speed, thereby suggesting that changes in processing also impact tradeoffs between speed and accuracy. However, the temporal resolution of fMRI is relatively low, usually on the order of seconds. Hence, methods are available for studying rapidly occurring neural activity (e.g., events measured in milliseconds) compared to fMRI.

EEG, unlike fMRI, offers temporal resolution but lacks spatial resolution. Numerous studies have shown that EEG can effectively track attention (Woodman and Luck, 1999; Heitz et al., 2010) and the temporal evolution of decision-making processes (Kelly and O’Connell, 2013; Van Vugt et al., 2014). In neuroscience, EEG can be utilized in three ways. The first involves recording EEG activity without stimuli, such as during sleep (Li et al., 2021). The second method involves capturing term stimuli like listening to music or watching continuous videos (Cong et al., 2013; Rogenmoser et al., 2016). The third approach focuses on event-related potentials (ERPs), which involve averaging trial EEG data and analyzing event-related oscillations (EROs) in the time-frequency domains based on the ERPs (Başar et al., 2001). Unlike the two methods mentioned earlier, event-related acquisition techniques allow researchers to investigate processes related to specific brain characteristics (Luck, 2014). Consequently, event-related EEG studies are gaining acceptance among researchers.

Numerous studies have explored the impact of speed accuracy on decision-making stages, such as sensory processing, mid decision and late motor processing. These studies have utilized an ERP component called lateralized readiness potentials (LRPs) (Osman et al., 2000; Van Der Lubbe et al., 2001; Rinkenauer et al., 2004; Wenzlaff et al., 2011). However, it is essential to note that there is still no conclusion on this topic (Heitz, 2014). This lack of clarity might be attributed to the simplicity of the task paradigm employed in studies and the challenges in distinguishing the decision-making process.

To address this gap, we have selected a dataset that focuses on mental rotation tasks, which are more challenging than the Franker paradigm, for extracting LRPs (Hoffmann and Falkenstein, 2010). As a result, participants require time to complete the rotation task, allowing for better differentiation between the early visual component and other components. In rotation tasks, participants were presented with two- or three-dimensional stimuli like images of objects, letters, or shapes (Shepard and Metzler, 1971; Cooperau and Shepard, 1973). Typically, during the phase of mental rotation, individuals process and understand the visual information from these stimuli in their cognitive system before making judgments and responding. Previous studies have observed that rotation-related negativity (an induced component with peaks around 300 ms) represents judgment regarding rotation (Heil and Rolke, 2002). However, our study examined the evoked components, P100 (P1) and N100 (N1) that occur before 200 milliseconds and are unaffected by the judgment process.

The P1 component, which appears as a positive deflection in the ERP waveform, occurs around 100 milliseconds after the stimulus is presented (Hillyard and Kutas, 1983). On the other hand, the N1 component, characterized by a negative deflection, typically happens approximately 140 milliseconds after the stimulus onset (Näätänen and Picton, 1987). These specific components were selected because they represent the sensory inputs in ERP (Luck et al., 1990). The P1 component is connected to the processing of information, including how we perceive spatial details in what we see (Russo et al., 2002). It is responsive to factors like attention and the demands of the task at hand (Hillyard et al., 1998). The amplitude of the P1 component induced by stimulation generated within the subject’s visual field is larger than that of the P1 component induced by stimulation outside the visual field. In other words, when individuals ignored the stimulus, the amplitude of the P1 component decreased. This highlights how attention plays a role in visual processing (Vogel and Luck, 2000). On the other hand, the N1 component is associated with the processing of visual stimuli and our sensory memory. It encodes aspects such as recognizing features and patterns while selectively attending to them (Hillyard et al., 1998). Factors like stimulus characteristics, individual variations, and experimental tasks can affect the amplitude and timing of N1. This underscores its significance in processing the characteristics of stimuli (Vogel and Luck, 2000).

When researchers extract ERP components, they often average trials to reduce noise. In addition to this practice, we use a toolbox of principal component analysis (PCA) along with Promax rotation to better extract our interested components (Zhang et al., 2020). In general, PCA is a method used to reduce the complexity of a dataset, which has been widely recognized for its ability to identify bioelectrical activity patterns while preserving information from origin effectively signals even when reducing data dimensions (Dien, 1998; Dien et al., 2005). In ERP research, PCA has been extensively used due to its effectiveness and relevance (Dien et al., 2007; Dien, 2012). Afterwards, we project the P1 and N1 components onto the field while considering any uncertainties regarding variance and polarity corrections. Finally, by combining spatial representations, we can selectively choose the components that are of interest while disregarding others.

However, the ERP signal only provides information about activity in the time domain, does not reveal any details about frequency. To address this limitation, we conducted a time-frequency analysis (TFA) on each ERP component we are interested in. This allowed us to extract event-related frequency features from the EEG signal. Based on Erol Basar’s theory, ERPs combine evoked oscillations with frequencies (Başar, 1998). According to Wolfgang Klimeschs, research suggests that the P1-N1 complex may be influenced by theta and alpha oscillations (Klimesch et al., 2004a). It is widely accepted that the theta band (4–8 Hz) is related to working memory while the alpha band (8–13 Hz) is associated with attention (Klimesch, 1999, 2012; Başar and Güntekin, 2008). It is generally accepted that EEG oscillations in the alpha band are particularly useful for studying attention. The amplitude of alpha band activity increases when attention is focused on external stimuli but decreases when attention is directed inwardly or when eyes are closed. In this study, we will analyze the evoked event-related oscillations (EROs) in the alpha and theta bands at different SAT instructions to determine if there were any distinctions between the two groups. We hypothesize that there will be variations in the attentional profiles, which could be detected by examining the power of the alpha band.

To extract evoked EROs, the conventional approach involves applying a short-time Fourier transform or wavelet transform to the ERP signal. This provides a time-frequency signal representation, allowing calculations within a region of interest. However, it is worth noting that evoked EROs have a shape resembling that of a waterdrop than a rectangle. Necessary information might be lost if the predetermined rectangular region is smaller than the waterdrop shape of the evoked EROs. On the other hand, irrelevant information could be included if the designated area is wider than the boundary of the evoked EROs. Additionally, relying on judgment for range selection hampers reproducibility in experiments. To address this issue and reduce subjectivity, we propose employing a range selection method called the Canny detector algorithm. John F. Canny created this algorithm in 1986 for edge detection, which will improve objectivity in image processing.

The Canny edge detection algorithm is widely employed in computer vision, image, and video processing (Yuan and Xu, 2015; Song et al., 2017; Sekehravani et al., 2020). Its primary purpose is to identify edges with intensity levels in an image (Canny, 1986). One of the advantages of using this algorithm is that it reduces the impact of factors introduced by researchers when labelling these edges. Since this ERO range extraction means has been proposed recently, it has yet to be widely used in time-frequency analysis. As a result, we have calculated the results of both methods in this study, hoping to understand the advantages and disadvantages of the two methods.

Overall, the present study sought to analyze the effects of different SAT instructions on subjects’ visual evoked ERP and ERO by applying various ERO extraction methods with a set of mental rotation EEG data. Early visually evoked ERPs and EROs can effectively represent subjects’ early perception, and we aimed to investigate the effects between different SAT instructions.

## 2 Materials and methods

Our study utilized a previously published dataset from experiments focusing on error-related potentials (Hoffmann and Falkenstein, 2015). Our study utilized a previously published dataset from experiments focusing on error-related potentials.

### 2.1 Participants and tasks

The dataset used in this study included 20 participants, of which 11 were female and 9 were male. The age range of the participants was between 21 and 27 years (mean = 23.8; SD = 1.9). The participants were randomly divided into two groups: one group was instructed to complete the task quickly (*N* = 13), while the other group was instructed to be precise (*N* = 7). The task was a mental rotation task modified for ERP measurement to yield a comparable timeline and workflow for the participants during the conduction of the experiment. One out of two letters (F, R) were presented to the participants. The letter was either rotated, mirrored across the central axis or both. Subjects had to indicate with a left or right button press of the corresponding thumb if the letter was mirrored or not. The letters were rotated by 5 degrees (0°, 45°, 135°, 225° or 315°). The 20 possible stimuli (5 × 2 × 2) were presented in random order. Since the type of letter did not affect the results of our study (Quan et al., 2017), we divided all the stimuli into five categories according to the angle of presentation of the stimuli.

In the task, the subjects received post-response feedback indicating whether they responded fast enough, too fast or too slow. The feedback consisted of two pictograms. If the participants responded fast enough, a yellow pictogram of a smiling face (“smiley”) appeared in the center of the screen. A red, angry-looking pictogram appeared if they responded too fast or too slow. Typically, subjects have decreased accuracy in rapid judgments. However, the study of this dataset was initially used to study error-related potentials. Hence, the deadline for the feedback was adapted block-wise. If the error rate in one block (80 trials) was below 8 %, the deadline was decreased, adding one standard deviation to the mean RT in the previous block. If the error rate was above 12%, the deadline was increased by adding four standard deviations to the mean RT of the previous block. Therefore, in this study, no behavioral analysis was performed.

### 2.2 Recording and pre-processing

EEG was recorded unipolar from 59 electrodes over frontal, central, parietal, occipital, and temporal areas (FPz, FP1, FP2, AFz, AF7, AF3, AF4, AF8, Fz, F7, F3, F4, F8, FCz, FT7, FC5, FC3, FC1, FC2, FC4, FC6, FT8, T7, C5, C3, Cz, C1, C2, C4, C6, T8, TP7, TP8, CPz, CP5, CP3, CP1, CP2, CP4, CP6, Pz, P7, P3, P1, P2, P4, P8, POz, PO9, PO7, PO3, PO4, PO8, PO10, Oz, O1, O2, M1, M2) and four electrooculographic (EOG) electrodes (SO2, IO2, LO1, LO2) from above and below the right eye. The data was pre-processed by using EEGLAB (sccn.ucsc.edu/EEG lab) (Delorme and Makeig, 2004) running on MATLAB (The MathWorks, Inc.) (Iversen and Makeig, 2019). In EEG pre-processing, a new reference was established using the calculated average of all the channels and downsampling the data to 150 Hz. Afterwards, the EEG data were filtered with a low cut-off frequency of 0.1 Hz, a high cut-off frequency of 20 Hz, and a notch frequency of 50 Hz. We used independent component analysis (ICA) on continuous data to eliminate components related to eye movements. To make the ICA decomposition run faster, we first down sampled the data to 100 Hz, and then we removed the data when subjects were at rest, i.e., the block-to-block data segments. During these times, subjects may be moving, chewing, etc., and these actions typically generate large voltages with inconsistent scalp distribution. We chose infomax-ICA as our ICA algorithm. After running ICA, we presented the horizontal and vertical electroocular signals (EOG) and then used the reconstructed signals for subsequent analysis. Furthermore, the signal from six electrodes (“HEOL,” “VEOD,” “HEOR,” “VEOU,” “M1,” and “M2”) were not analyzed further. In the subsequent analysis, we focused on four occipital electrodes, P7, P8, PO7, and PO8, which, according to previous studies, best represent the visual component (Russo et al., 2002; Creel, 2019).

Subsequently, the continuous EEG data were divided into several epochs (trials) depending on the timing from 200 ms before to 300 ms after stimulus onset. The baseline correction was accomplished by subtracting the baseline period’s (from −200 to 0 ms) mean amplitude for all time points. Bad trials include error trials, and timeout trials were rejected (about 80 trials were reserved for each participant’s stimulation).

### 2.3 Temporal ERP analysis

In present study, we compared two ERP score methods, and we made the traditional method M1, in which we analyzed the amplitude of the P1 component and the N1 component. After preprocessing the ERP signals, we scored P1 amplitude between 80 and 120 ms and N1 amplitude between 140 and 190 ms, expressed as M1-A1 and M1-A2, respectively.

**Figure 1:**
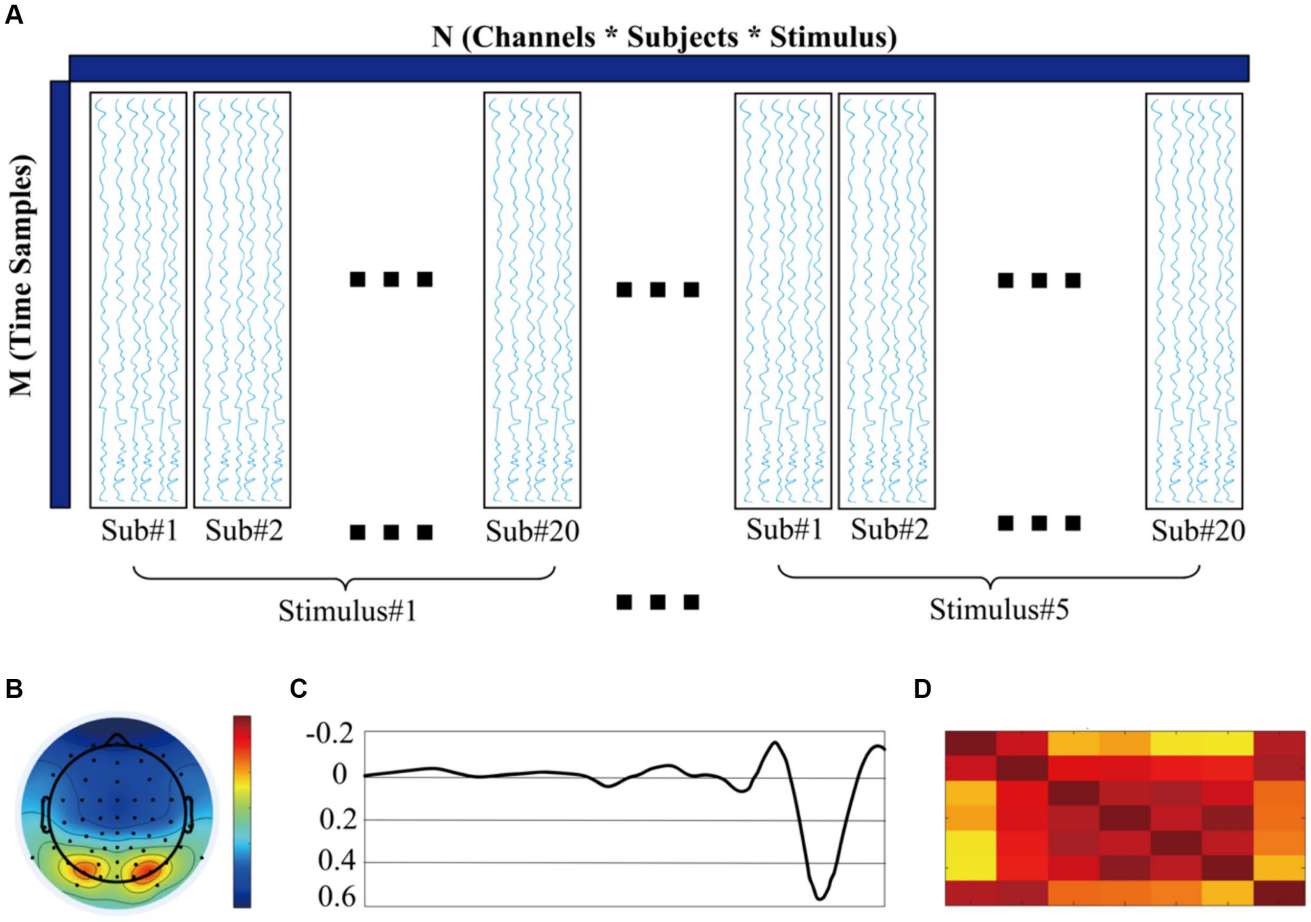
**(A)** ERP data matrix for temporal principal component analysis. **(B)** Spatial representation of P1 components (topography). **(C)** Temporal representation of P1 components. **(D)** Similarity of P1 components across subjects.

In the second method, we applied temporal principal component analysis to extract the P1 and N1 components, respectively. For the brevity of expression, this approach is subsequently referred to as M2. To begin with, we stored the preprocessed data in a fourth-order tensor with the index name of channel^∗^time points^∗^stimuli^∗^participants. For this study, the fourth-order tensor is 59^∗^75^∗^5^∗^20, representing 59 channels and 75 time points within each epoch. The group factors include five rotation angle levels (0°, 45°, 135°, 225°, 315°) and contain 20 subjects (13 subjects from the fast group and the other seven subjects from the precise group). Second, we apply tPCA and Promax rotation to decompose this ERP data matrix $\hat{Z}=Z^{T}\in R^{N\times M}$. Here, N denotes the number of sensors across all conditions and subjects within the tensor, while M represents the time points within one epoch. Refer to Figure 1 for representation. It is worth noting that time points are the main component in matrix Z. Other factors, such as channel, condition, and subject, are integrated into the row marked as other components. By utilizing tPCA and Promax rotation techniques, we decomposed this ERP tensor into 23 components, which explained 99% variance of the original data. To resolve variance and polarity uncertainties, we back-projected each component into the time and space. As for P1, we select components with peaks between 80 and 120 ms and N1 between 140 and 200 ms. In the last step, we back-project our chosen components into the time domain. Afterward, we scored P1 amplitude between 80 and 120 ms and N1 amplitude between 140 and 190 ms, expressed as M2-A1 and M2-A2, respectively. All amplitude calculations were performed in the MATLAB-based toolbox ERP_ERO_v1.1.

### 2.4 Time-frequency analysis

In this study, we compare two approaches, for extracting ERO. We will refer to the method as M1, the conventional approach, and the second method as M2, a more recently developed approach. In the first method, we directly apply continuous wavelet transform to the time-frequency representation of the filtered ERP data. The frequency range of interest is set between 0.5 and 30 Hz, with 30 frequency bins distributed nonlinearly. We also set the center frequency and bandwidth to 1. For each layer, we correct the power values by subtracting the mean baseline power (measured 200 ms before stimulus onset) at each point (Hu et al., 2014; Benvenuti et al., 2017; Peng et al., 2019). To determine our areas of interest using this method, we extract alpha power (8–13 Hz) during the P1 period and theta power (4–8 Hz) during the N1 period using a method. These areas are denoted as M1-R1 and M1-R2, respectively.

The second method of ERO extraction was proposed by Zhang et al. (2020). The main idea involves using tPCA with Promax rotation to identify the ERP components of interest before conducting time-frequency analysis (in the following text, we will refer to this method as PCA-TFA). This approach helps differentiate the components in terms of time and space, minimizing any interference caused by overlapping components.

We have described the steps of PCA in the introduction of time analysis of ERP. On the basis of the ERP analysis, we performed wavelet transforms to convert the P1 and N1 components after PCA processing into time-frequency representations. For comparison, we used the same wavelet transform parameters as the conventional method to obtain the time-frequency representation of the P1 and N1. Following this, baseline correction was achieved by subtracting the mean power of the baseline (200 milliseconds before the stimulus onset) from the values of each point in the time-frequency representation.

### 2.5 Determining evoked EROs region via edge detection

**Figure 2:**
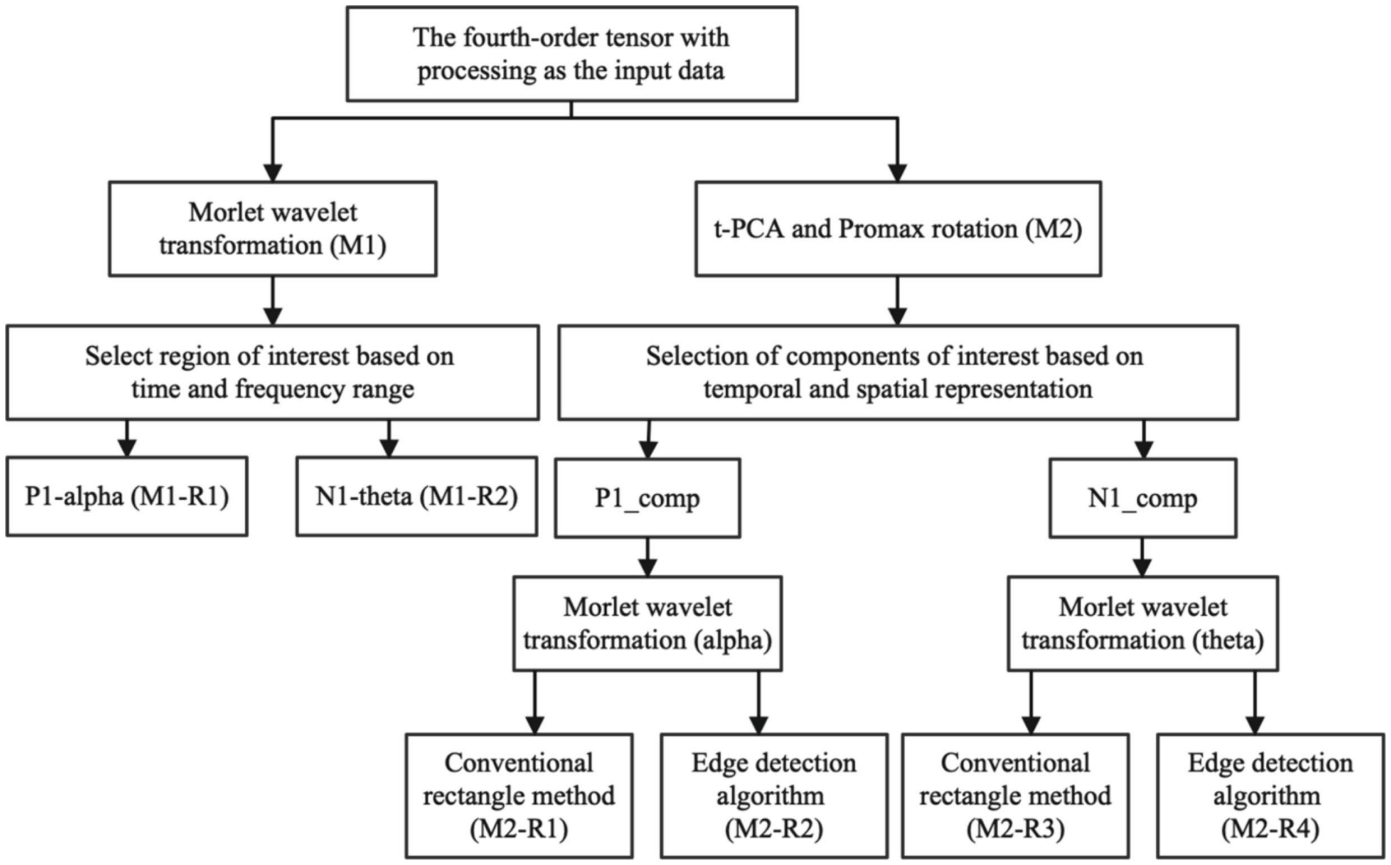
In the ERO extraction method, we input processed fourth-order tensor ERP data. M1 represents the conventional Evoked ERO time-frequency analysis approach, while M2 refers to the method that involves tPCA and Promax rotation before conducting the time-frequency analysis. In the conventional method, M1-R1 indicates the power in the P1 alpha band, and M1-R2 represents the power in the N1 theta band, also extracted using rectangles. On the other hand, in the method involving tPCA and Promax rotation, M2-R1 denotes the ERO of the P1 alpha band obtained through rectangular extraction, and M2-R2 signifies the ERO of the P1 alpha band derived via the Canny edge detection algorithm, M2-R3 stands for N1 theta bands ERO extracted using traditional rectangular extraction, and M2-R4 represents N1 theta bands ERO obtained through Canny edge detection algorithm.

As mentioned earlier in the section, the conventional rectangular method is commonly utilized to identify the region in time-frequency analysis. However, this method is based on the subjective judgment of the experimenter, which leads to less reproducibility. Hence, for ERO extraction in our study, we employed an algorithm based on edge detection. In our approach, we utilized the edge detection algorithm to determine the edge strength by calculating the amplitude and direction of gradients at each location in the time-frequency representation after averaging across subjects for each condition. This involved finding the amplitude by examining the direction for each pixel among eight possible primary directions (i.e., 0, 45, 90, 135, 180, 225, 270, and 315 degrees). If a pixel’s gradient amplitude was more significant than its two neighboring pixels along that direction, it was retained; otherwise, it was set to zero. A range of EROs was calculated by specifying a fixed threshold, and then the evoked ERO for each subject in each condition was extracted based on the calculated teardrop circle. The specific calculation steps are shown in Figure 2.

Finally, we used two-way repeated-measurement-ANOVA (rmANOVA) with five levels of rotation degree as within-subject factors and two different SAT groups as between-subject factors for analysis (Table 1).

**Table 1:** Event related potentials results.

Amplitude	Angle			Group			Angle × group		
	*F*	*P*	$\eta_{p}^{2}$	*F*	*P*	$\eta_{p}^{2}$	*F*	*P*	$\eta_{p}^{2}$
M1-A1	1.122	0.353	0.059	3.101	0.095	0.147	0.736	0.570	0.039
M1-A2	14.083	**<0.001**	0.439	0.222	0.643	0.012	0.880	0.480	0.047
M2-A1	0.685	0.605	0.037	6.679	**0.019**	0.271	0.494	0.740	0.027
M2-A2	14.362	**<0.001**	0.444	0.219	0.646	0.012	0.905	0.466	0.048

*P*-values where significance exists have been bolded in the table.

## 3 Results

### 3.1 Event related potentials results

**Figure 3:**
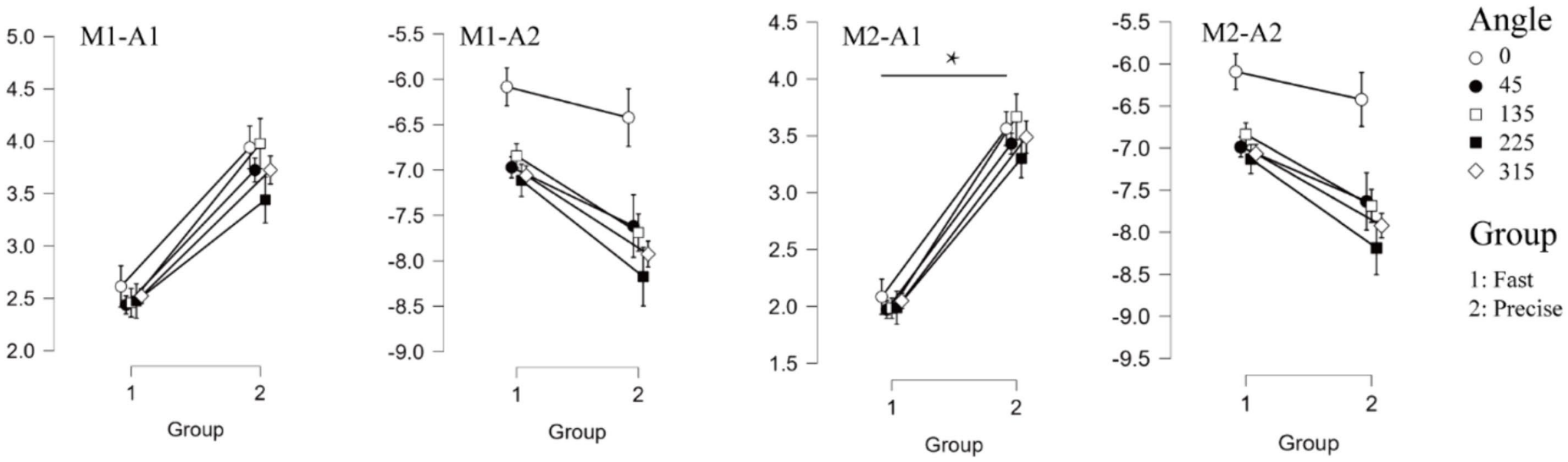
Event related potentials results. The four graphs on the top represent the results for the amplitude of the two components (M1-A1, M1-A2, M2-A1, M2-A2 respectively). Where “*” indicates a significant difference between the two groups.

For the mean amplitude analysis of the ERP, the traditional analysis method showed that no significant differences were found between the two groups (*F* = 3.101, *p* = 0.095, *η_p_*^2^ = 0.147 for P1 and *F* = 0.222, *p* = 0.634, *η_p_*^2^ = 0.012 for N1). The results of the tPCA-based method, on the other hand, showed that the mean amplitude of P1 for the two groups, was significantly different, with the mean amplitude of P1 for the subjects who were required to respond quickly being significantly smaller than for the subjects who were required to respond accurately (*F* = 6.679, *p* = 0.019, *η_p_*^2^ = 0.271). Both analyses methods similarly found that there was a significant difference between within-group analyses for the N1 component (*F* = 14.083, *p* < 0.001, *η_p_*^2^ = 0.439 for M1 and *F* = 14.362, *p* < 0.001, *η_p_*^2^ = 0.444 for M2). Both methods were found to interact for amplitude (Figure 3).

### 3.2 Event related oscillations results

**Figure 4:**
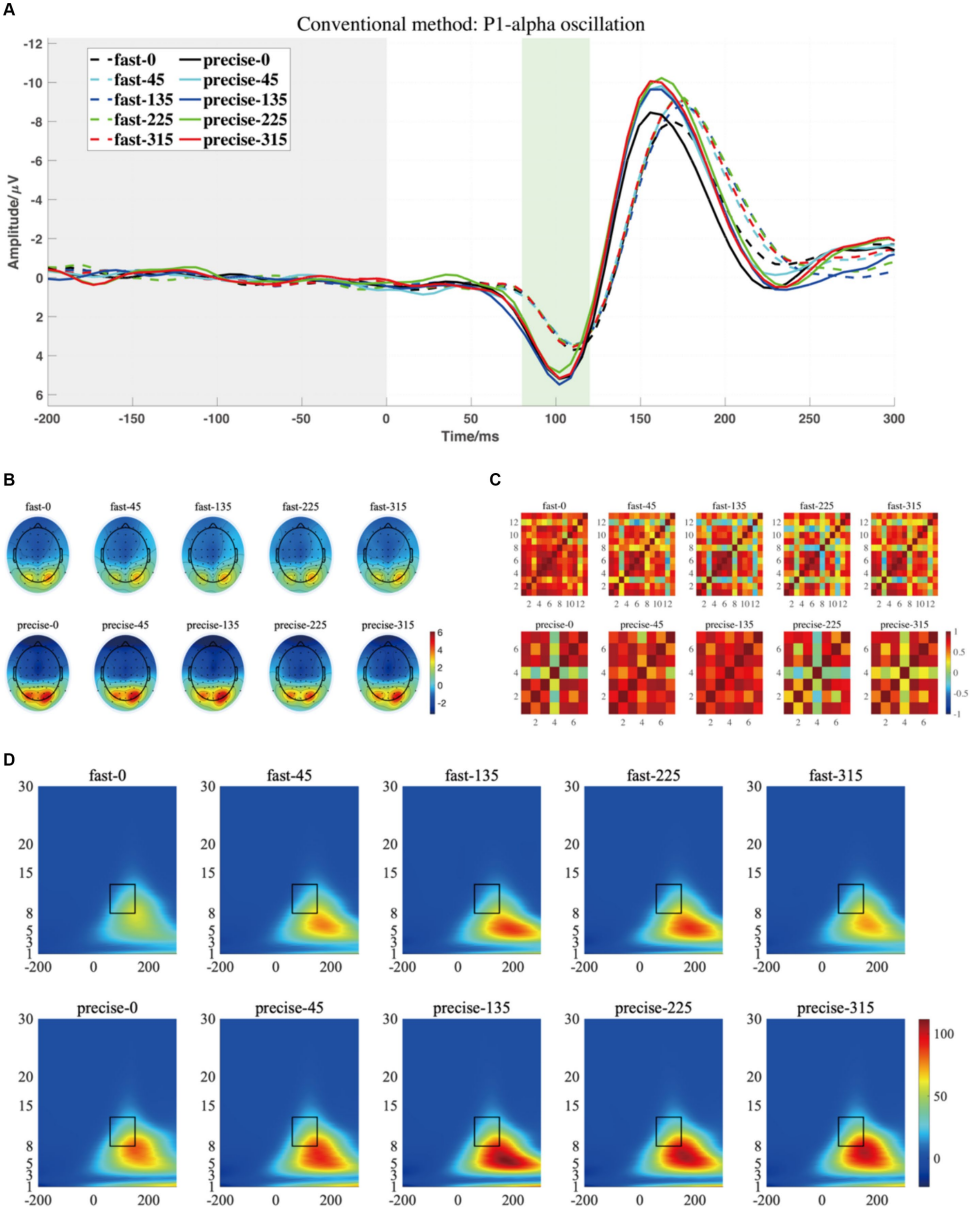
**(A)** The grand averaged waveform (at P7, P8, PO7, and PO8 electrodes) where the grey area is the baseline component and the position of the horizontal coordinate 0 is the stimulus presentation time. **(B)** The topography has a time window from 80 to 120 ms (the green area). **(C)** The similarity of topographies across participants of each group and condition for the filtered data. **(D)** The grand averaged time-frequency representation (TFR) of every group and condition. The black rectangular box in the figure marks the P1-alpha evoked ERO extracted by the traditional method with a 60–150 ms time window and a frequency of 8–13 Hz, referred to as M1-R1.

**Figure 5:**
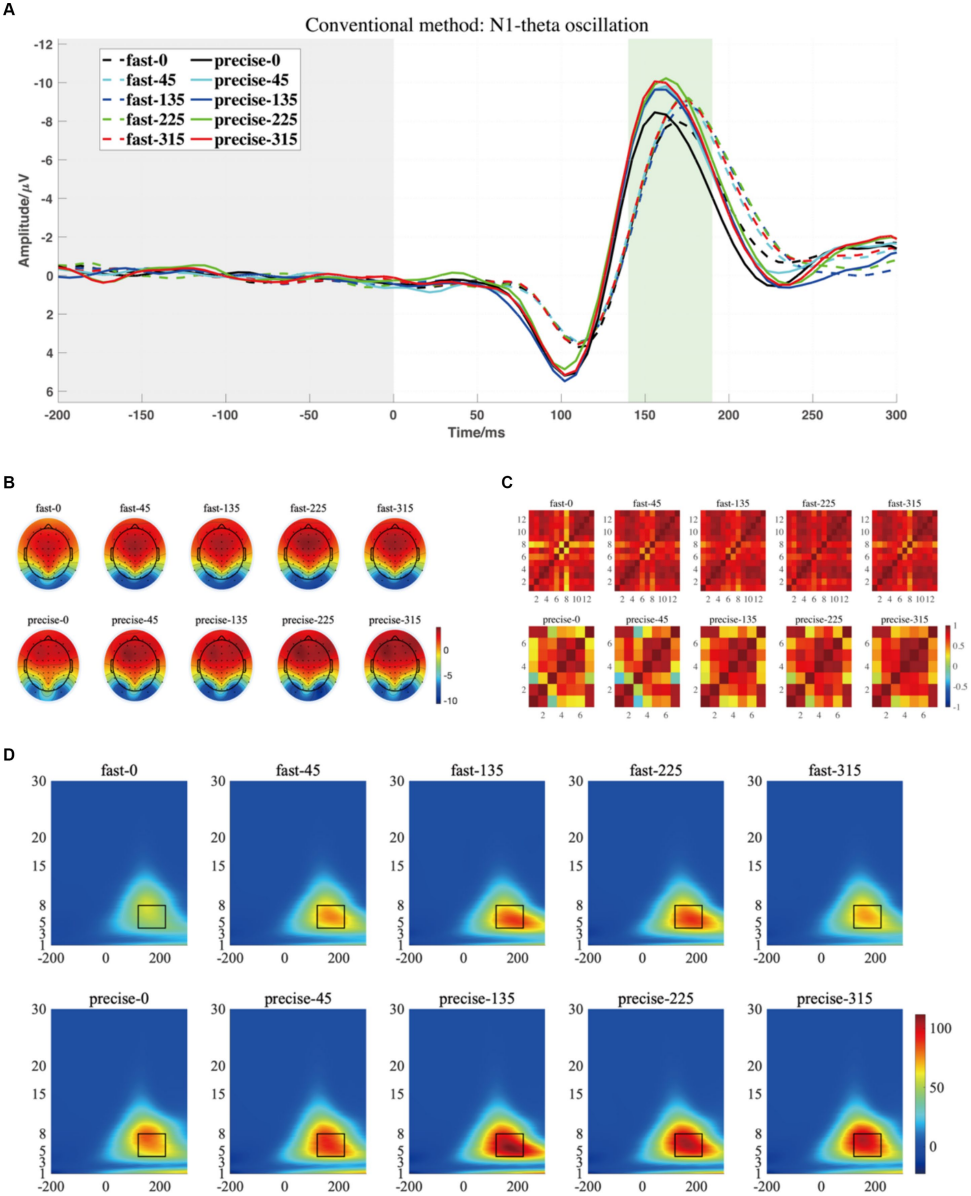
**(A)** The grand averaged waveform (at P7, P8, PO7, and PO8 electrodes) where the grey area is the baseline component and the position of the horizontal coordinate 0 is the stimulus presentation time. **(B)** The topography has a time window from 140 to 190 ms. **(C)** The similarity of topographies across participants of each group and condition for the filtered data. **(D)** The grand averaged time-frequency representation (TFR) of every group and condition. The black rectangular box in the figure marks the N1-theta evoked ERO extracted by the traditional method with a time window of 120–220 ms and a frequency of 4–8 Hz, referred to as M1-R2.

**Figure 6:**
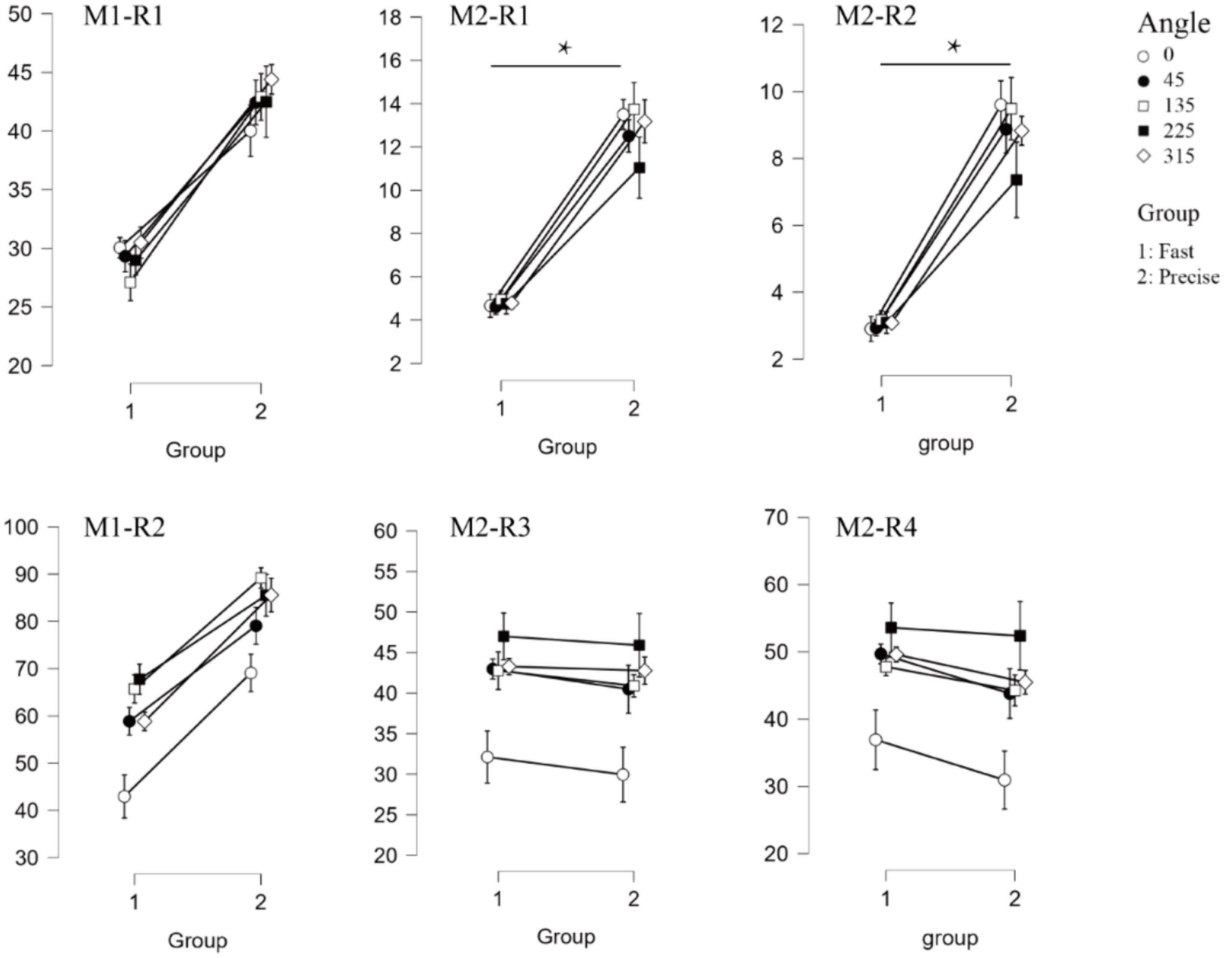
Oscillatory results associated with the event. The top three panels show the results of the P1-alpha oscillations (M1-R1, M2-R1, M2-R2, respectively). The bottom three panels show the results of N1-theta oscillations (M1-R2, M2-R3, M2-R4, respectively). Where “*” indicates a significant difference between the two groups.

For P1-alpha and N1-theta oscillation extracted using conventional time-frequency analysis methods in Figures 4D, 5D, the statistical result of the two EROs regions demonstrated that no significant differences were found for either comparison between SAT groups or interactions, as shown in M1-R1 in Table 2 and M1-R2 in Table 3. As for the analysis at N1-theta, there was a significant difference in the within-group analysis, as shown by M1-R2 in Table 3 and Figure 6. Subjects had significantly less energy in N1-theta at a rotation angle of 0 than at the remaining angles. In addition, in the time-frequency diagram of the conventional method, the primary energy is concentrated around the 4 Hz band around 180 ms. Since the energy in the early alpha band was not identified when the edge detection algorithm was used in conventional time-frequency analysis results, it was not further analyzed.

**Table 2:** The statistical result of P1-alpha oscillation for conventional time-frequency analysis (“M1”) and PCA-TFA (“M2”).

ROI	Angle			Group			Angle × group		
	*F*	*P*	$\eta_{p}^{2}$	*F*	*P*	$\eta_{p}^{2}$	*F*	*P*	$\eta_{p}^{2}$
M1-R1	0.832	0.509	0.044	2.315	0.143	0.115	0.792	0.534	0.042
M2-R1	1.434	0.232	0.074	7.21	**0.015**	0.286	1.246	0.299	0.065
M2-R2	1.546	0.198	0.079	6.299	**0.022**	0.259	1.734	0.152	0.088

**Table 3:** The statistical result of N1-theta oscillation for conventional time-frequency analysis (“M1”) and PCA-TFA (“M2”).

ROI	Angle			Group			Angle × group		
	*F*	*P*	$\eta_{p}^{2}$	*F*	*P*	$\eta_{p}^{2}$	*F*	*P*	$\eta_{p}^{2}$
M1-R2	11.222	**0.002**	0.384	0.552	0.698	0.030	0.552	0.365	0.046
M2-R3	9.212	**<0.001**	0.339	6.08E-05	0.994	3.38E-06	0.076	0.989	0.004
M2-R4	9.177	**<0.001**	0.338	0.044	0.835	0.002	0.181	0.948	0.01

**Figure 7:**
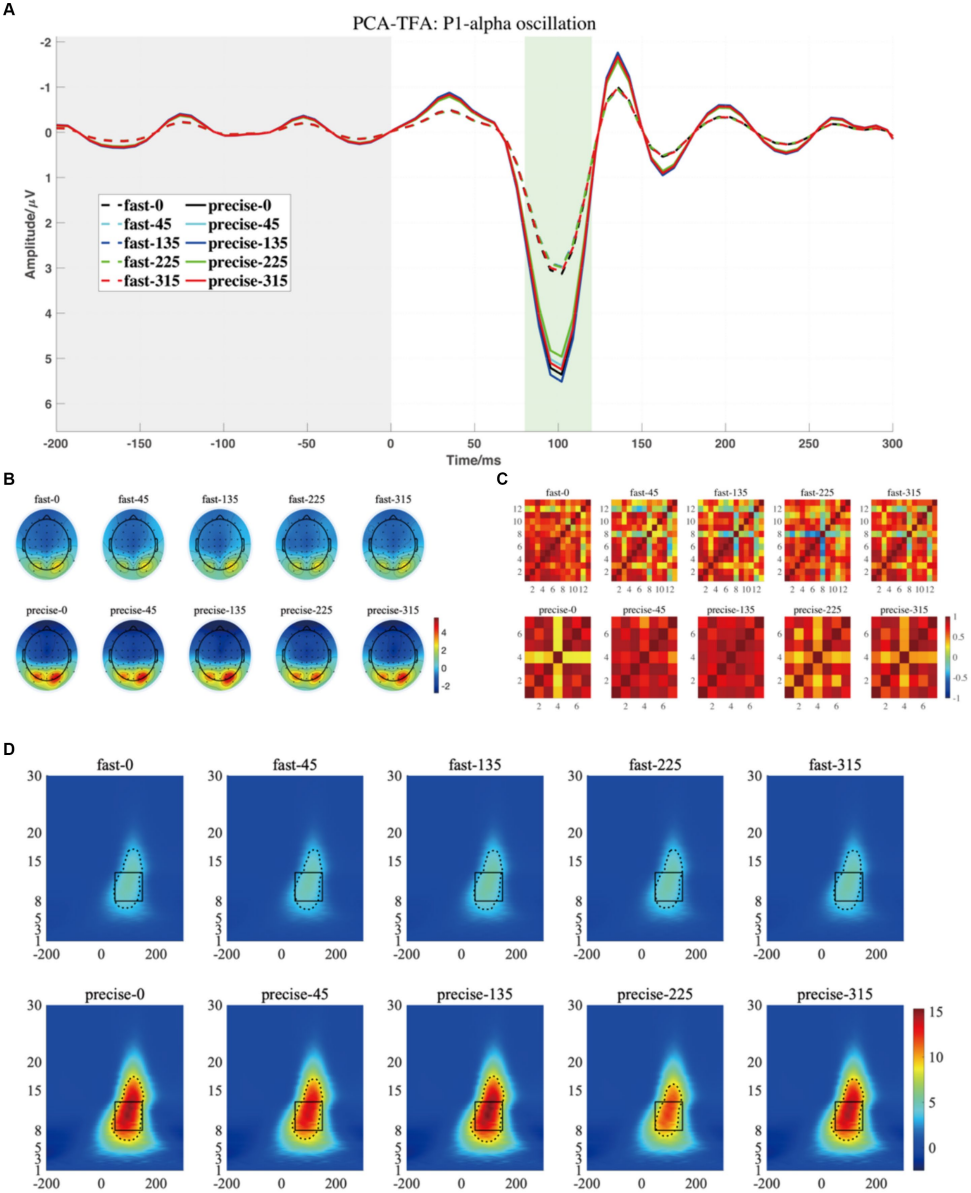
**(A)** The projected waveform of P1 components (at P7, P8, PO7, and PO8 electrodes) where the grey area is the baseline component and the position of the horizontal coordinate 0 is the stimulus presentation time. **(B)** The topography has a time window from 80–120 ms. **(C)** The similarity of topographies across participants of each group and condition for the projected data. **(D)** The grand averaged time-frequency representation (TFR) of every group and condition. The black rectangular box in the figure marks the P1-alpha evoked ERO extracted by PCA-TFA with a 50–150 ms time window and a frequency of 8–13 Hz, M2-R1. The dashed line is the range extracted using the Canny algorithm, which is M2-R2.

**Figure 8:**
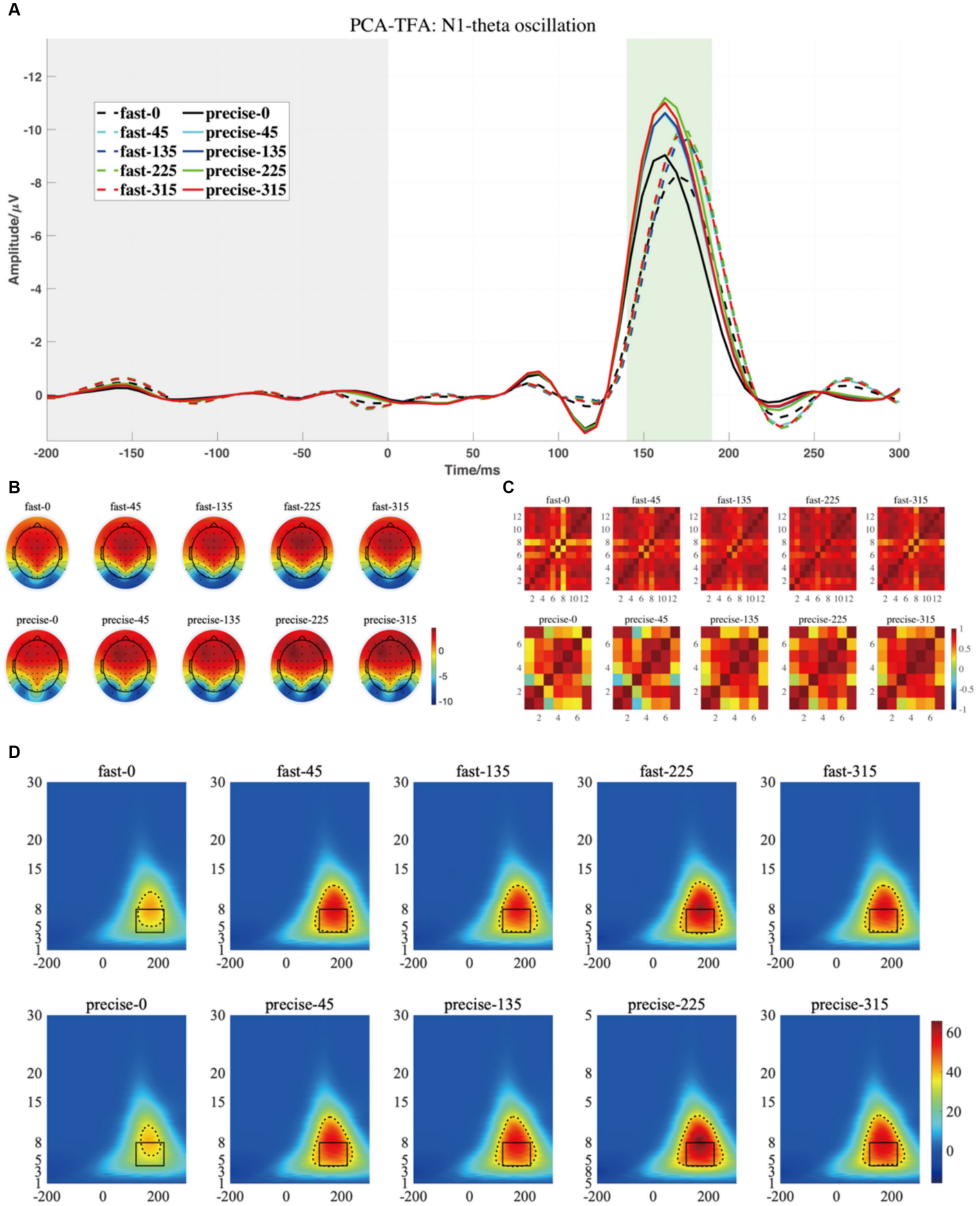
**(A)** The projected waveform of N1 components (at P7, P8, PO7, and PO8 electrodes) where the grey area is the baseline component and the position of the horizontal coordinate 0 is the stimulus presentation time. **(B)** The topography has a time window from 140–190 ms. **(C)** The similarity of topographies across participants of each group and condition for the projected data. **(D)** The grand averaged time-frequency representation (TFR) of every group and condition. The black rectangular box in the figure marks the N1-theta evoked ERO extracted by PCA-TFA with a time window of 120–220 ms and a frequency of 4–8 Hz, M2-R3. The dashed line is the range extracted using the Canny algorithm, which is M2-R4.

Figures 7, 8 depict the projected waveforms of some occipital electrodes, the topographic distribution in the time domain, the associated topographic similarity between the two groups of subjects, and the TFR in each case for P1-alpha and N1-theta. We retained 23 components in the principal components analysis, which accounted for 99% of the variance.

Based on the temporal and spatial characteristics of the P1 component, we selected the seventh component for further analysis, each of which explains 0.97% of the variance. By comparing the temporal waveforms in Figures 4A,B, 7A,B, the back-projected waveforms and topography are consistent with the conventional grand-average waveforms in evolution and trend. Moreover, the spatial similarity of Figure 7C indicates minimal variability in the data across subjects, thereby attesting to its high quality. As for the statistical results, we found significant differences between the two groups, either using the conventional rectangular box method or the edge detection algorithm (Table 2, “M2-R1” and “M2-R2”). suggesting that subjects had less energy in the P1-alpha band when making fast judgments (*F* = 7.21, *p* = 0.015, *η_p_*^2^ = 0.286 for the conventional rectangular method and *F* = 6.299, *p* = 0.022, *η_p_*^2^ = 0.259 for the edge detection algorithm). In addition, we did not find significant differences between angles or interactions of groups and angles in within subjects’ analysis of P1-alpha.

Similarly, N1, the third and the eighth components were selected and projected back to the electrode fields, and the two components explain 0.65 and 5.61% of the variance, as shown in Figure 8A. We then used wavelet transform to compute the TFRs of the back-projected waveform. Similar to the results produced by the conventional method, the N1-theta power shows significant differences at different angles, regardless of the range intercepted using the conventional rectangular method or the edge detection algorithm (Table 3, M2-R3 and M2-R4). As can be seen in Figure 8D, the energy at 0 degrees is significantly smaller than at other angles (conventional rectangular range: *F* = 9.212, *p* < 0.001, fang = 0.339, edge detection algorithm: *F* = 9.177, *p* < 0.001, fang = 0.338). However, no significant differences were found in the comparisons and interactions between the different SAT groups.

## 4 Discussion

The current study investigated the effect of different speed-accuracy instructions on subjects’ perceptual process. As mentioned in the literature review, we chose a dataset that used a more difficult task, mental rotation, which requires more cognitive engagement than the lateral discrimination task (Hoffmann and Falkenstein, 2010). In the experiment, subjects were asked to determine if the letters were mirrored, and the two groups of subjects were guided by verbal instructions to respond quickly and accurately. In this study, we used principal component analysis to downscale the data. We selected the component of interest, P1 and N1 visually evoked component, and combined the P1 evoked ERP and alpha ERO and N1 ERP and theta ERO to evaluate subjects’ perception. The results of this study show that fast or accurate instructions changed subjects’ perceptual process during decision-making, as evidenced by early visual components.

The decision-making process usually includes several steps: identifying the problem, generating alternatives, evaluating alternatives, choosing an alternative, implementing the decision, and evaluating decision effectiveness (Lunenburg, 2010). In tasks where visual information is used as a stimulus, ‘identifying the problem’ usually involves processing visual information. Research has shown that visual information is critical for attention and judgment during decision-making (Padilla et al., 2018) To ensure accurate and efficient decision-making outcomes, participants must direct their attention using appropriate visual coding techniques to identify the required vital information (Eberhard, 2023).

In the current study, we focused on the early visual P1 and N1 components since P1 and N1 are two early components of opposite polarity, suggesting that neural synchrony occurs within a short time window and may alternate between inhibitory and excitatory (Klimesch et al., 2004b). Previous research has established that the P1 component suppresses irrelevant information, while the N1 component is associated with processing information of concern. In other words, P1 reflects the inhibitory process, while N1 reflects the excitatory process (Hillyard et al., 1994). Based on Klimesch’s theoretical framework, the P1-N1 component is generated by the simultaneous activation of three neuronal network systems, namely working memory, attention, and semantic memory systems, each with different frequency information, the frequency information of the three systems being focused on theta (about 6 Hz), the lower alpha (about 8 Hz) and the upper alpha (about 12 Hz) (Klimesch et al., 2004b). Whereas in our chosen method, the time-frequency representations of P1 and N1 are extracted separately by applying wavelet transforms to each of the two components, we find that the energy of P1 is mainly concentrated in the alpha band. In contrast, the energy of N1 is mainly concentrated in the theta band, which is the same as that of Gruber’s result (Gruber et al., 2005). In Gruber’s study the contribution of alpha and theta band energies to P1 and N1 was revealed. For alpha, the effect on P1 is always greater than that on N1, while for theta, the effect on N1 is always greater than that on P1. This is the theoretical underpinning of our main study of P1-alpha ERO and N1-theta ERO.

The current study observed a significant difference in amplitudes and α-ERO of the P1 component between the speed- and accuracy-emphasis groups. Specifically, the speed-emphasis group exhibited lower α-ERO at the visual electrode sites than the accuracy-emphasis group. This finding is consistent with the notion that decreased alpha-band energy is associated with increased attentional allocation (Foxe and Snyder, 2011). Several studies have shown that alpha oscillations are modulated top-down when subjects allocate attention while being modulated by attention. Whereas the specific task influences the enhancement or weakening of alpha oscillations, some studies have confirmed that alpha power in the visual cortex decreases with increasing attention when attention is directed toward external visual events (Worden et al., 2000; Sauseng et al., 2005; Rajagovindan and Ding, 2011); when alpha power increases with attention when attention is directed to internal representations, such as during visual imagery and working memory retention. It is worth noting that although the visual imagery task was chosen for the present study, the early evoked components of attention, P1 and N1, were evoked with picture stimuli and thus were external visual events. When using the traditional TFA method for extracting early alpha features, we found no difference between the two groups of subjects. However, when calculating the alpha band energy of the P1 component separately using the PCA-TFA method, it was found that the subjects required to respond quickly had significantly less energy than those required to respond more accurately.

Also, in the comparison of the amplitudes of the P1 components, we found differences between the two methods. In the traditional analysis method, the amplitude of P1, no significant difference was found between the two groups, whereas after the tPCA treatment, it was found that the amplitude of subjects when asked to respond quickly was significantly smaller than that of subjects who were asked to respond accurately. The amplitude and alpha oscillations of the P1 component, which produce similar results, can be explained by ERP-oscillation theory. The main reason for the difference between the two time-frequency analysis methods may be that PCA pre-processing helps to remove noise and other irrelevant signals. For example, when only the P1 component is studied, the N1 component is equivalent to a noisy signal. It has been demonstrated that PCA can help interpret the structure of ERP datasets, allowing researchers to select components of interest for subsequent analysis according to the experimental purpose (Dien et al., 2007; Dien, 2012).

In our study, subjects who were asked to respond quickly were found to have less P1-alpha power than those who were asked to be accurate. According to previous research, Alpha-band oscillations as evidence for an attention-mediated mechanism of selective suppression of interfering information (Pfurtscheller et al., 1996; Klimesch, 2012). Specifically, higher alpha power indicates lower cortical excitability and higher perceptual thresholds. This is true for both naturally occurring and task-related alpha band activity in visual cortex. Therefore, we suppose that different speed-accuracy instruction words did influence participants’ attentive profile, with those who were required to respond quickly spending more attention on the task. That may be because the emphasis on accuracy may have led to lower tension in participants, resulting in lower levels of attention than the group that was required to respond quickly. We, therefore, suggest that the different SAT strategies chosen by subjects should be fully considered in cognitive-behavioral experiments and that researchers should be fully aware of the effects of the cognitive processes of interest in the study on response times and correctness. For example, when studying the change-detection task, subjects must determine whether two consecutive visual objects are identical or whether the latter object has changed. In this type of task, the researcher is interested in the subject’s working memory capacity, which primarily affects the correctness of the task. Therefore, the guide should be chosen so the subject can respond as accurately as possible. However, suppose the researcher is interested in the efficiency between the working memory entry and the test display. In that case, the main concern should be the subject’s response time, so the guide should allow the subject to respond as quickly as possible.

Interestingly, no significant difference was found between the two groups regarding the amplitudes and theta ERO of the N1 component. However, significant differences within each group were observed, with the N1 component amplitudes and ERO being significantly smaller at a 0-degree rotation in the mental rotation task compared to other angles (45, 135, 225, and 315 degrees). This result might be attributed to the role of the N1 component in discrimination tasks, as suggested by Luck and Hillyard (1994) and Luck et al. (2000). The N1 component is closely related to the automatic processing of visual stimuli, sensory memory, feature detection, pattern recognition, and selective attention (Gratton et al., 1988). In contrast, the increased difficulty of discrimination for stimuli at other angles (45, 135, 225, and 315 degrees) might demand more cognitive resources for task completion. Thus, the significantly smaller N1 component amplitudes and ERO at a 0-degree rotation compared to other angles in the mental rotation task could result from the differences in cognitive resources required for discrimination tasks at various angles. In other words, the 0-degree stimuli might be more straightforward to discriminate and require fewer cognitive resources. This finding further elucidates the role of the N1 component in visual discrimination tasks and highlights the influence of task difficulty on neural activity (Mangun and Hillyard, 1991; Vogel and Luck, 2000). Moreover, it provides novel insights into the neurophysiological mechanisms underlying human decision-making under different task demands.

In addition, the traditional rectangular approach and the Canny edge detection algorithm-based approach were compared when determining the evoked ERO regions. The Canny edge detection algorithm is widely used in computer vision and medical image processing. Furthermore, the time-frequency analysis picture can be interpreted as a two-dimensional matrix, which means we can also detect edges with the Canny algorithm. In this study, the traditional rectangular range selection method and canny algorithm got the same result, as shown in Tables 2, 3. Previous studies also showed that the two methods got different results. The Canny algorithm would find significant differences that the traditional method could not detect (Zhang et al., 2020). This contradictory finding is likely to be related to the difference in the data. In other words, the Canny algorithm extracts ERO ranges that are closer to the reality of the situation, like the shape of a waterdrop, whereas the traditional rectangular approach compares the energies within a fixed rectangular range under different conditions. If the data comparison process produces differences that are too pronounced, then both methods will produce similar results. However, the edge detection algorithm also has some limitations compared to the traditional rectangular box approach. For example, if edge detection algorithm is used directly to identify ERO regions from the TFR of the averaged ERP data, a region may involve different spatially distinct oscillations when these components overlap in time. Such like in Figure 8D, the range based on edge detection is approximately 5–16 Hz, which exceeds the general frequency range of alpha (8–13 Hz). In other words, using the PCA-TFA approach, the components of interest can be better differentiated temporally. However, a fixed frequency range cannot be selected. Therefore, the decision between utilizing traditional rectangular methods or edge detection algorithms requires a case-by-case analysis based on the specifics of the research content.

In our study, several limitations should be considered when interpreting the results. Firstly, the sample size needed to be bigger, which might limit the generalizability of our findings. We next intend to recruit a larger sample to increase the external validity of the results. Furthermore, the dataset utilized in this study involved a task in which only different SAT guiding phrases were used to influence participants’ speed-accuracy trade-offs, and the deadline was adjusted according to their accuracy rates. As a result, examining the differences in behavioral data or latency was meaningless. Future research may consider suitable task designs to investigate the relationship between SATs and behavioral outcomes.

Regarding the future investigations of neural difference between SATs, it can be carried out from the following aspects. Firstly, we merely analyzed the evoked oscillations from the averaged ERP as mentioned above. It should be noted that some significant information like induced oscillations was cancelled out by the averaging over trials in the time domain. Induced oscillations are usually produced by nonlinear mechanisms or autonomous mechanisms and are high-order processes, such as the late component P3, which is often used to study excitatory and inhibitory mechanisms. The evoked oscillation is related to the stimulus lock-in time. Follow-up research can focus on the differences in induced components under different speed accuracy, but what needs to be paid attention to is the choice of paradigm. For example, the P3 component is usually elicited using the oddball paradigm. Secondly, there are many ways to adjust speed and accuracy, such as the instructions studied in this article to regulate the subjects’ SAT, and other methods such as whether there is a deadline, reward and punishment mechanisms, etc. There is currently no unified understanding of the regulatory mechanisms of different SAT regulation methods on subjects’ cognitive level. Our study shows that different speed accuracy guides, the most used method, influence subjects’ early event-related oscillations. Even though there were no differences in the behavioral data of the subjects during the experiment due to the experimental design. In summary, combining neuroscience methods with SAT methodology may provide a deeper understanding of the brain processes that underlie decision-making. There is growing agreement that SAT is a complex phenomenon that affects many aspects of decision-making and is associated with unique alterations in brain activity.

## Data availability statement

Publicly available datasets were analyzed in this study. This data can be found at: https://dataverse.harvard.edu/dataset.xhtml?persistentId=doi:10.7910/DVN/XHANW7.

## Author contributions

HL: Writing – original draft. XW: Methodology, Software, Writing – review & editing. TH: Funding acquisition, Supervision, Writing – review & editing. ZM: Supervision, Writing – review & editing.
